# Possible Pediatric Autoimmune Neuropsychiatric Disorders Associated With Streptococcal Infections (PANDAS) Presenting in Late Adolescence and Early Adulthood: A Case Series

**DOI:** 10.1002/ccr3.73768

**Published:** 2026-10-23

**Authors:** Tariq A. Madani, Fahd S. Alsallum, Ola H. Abudaowd, Salman T. Madani

**Affiliations:** ^1^ Division of Infectious Diseases, Department of Medicine, Faculty of Medicine King Abdulaziz University, and King Abdulaziz University Hospital Jeddah Saudi Arabia

**Keywords:** obsessive‐compulsive disorder, pediatric autoimmune neuropsychiatric disorders associated with streptococcal infections (PANDAS), *Streptococcus pyogenes*, tics, young adults adolescence

## Abstract

PANDAS‐like syndromes may rarely occur beyond childhood. Although these patients may meet criteria for PANS, recognizing a possible streptococcal‐triggered subgroup remains important for diagnosis, future biomarker discovery, and refinement of disease classification. Clinicians should consider post‐infectious autoimmune neuropsychiatric syndromes in older patients with abrupt‐onset OCD or tics following pharyngeal illness.

**Abbreviations**
ANA: antinuclear antibody
Anti‐AMPAR 1/2: anti‐α‐amino‐3‐hydroxy‐5‐methyl‐4‐isoxazolepropionic acid receptor 1/2
Anti‐CASPR2: anti‐contactin‐associated protein‐like 2
Anti‐GABABR: anti‐gamma‐aminobutyric acid type B receptor
Anti‐GAD: anti‐glutamic acid decarboxylase
Anti‐LGI1: anti‐leucine‐rich glioma‐inactivated 1
Anti‐MBP: anti‐myelin basic protein
Anti‐NMDAR: anti‐N‐methyl‐D‐aspartate receptor
Anti‐PNMA2 Ma2/Ta: anti‐purkinje cell nuclear antigen 2 Ma2/Ta
ARFID: avoidant/restrictive food intake disorder
ASO: antistreptolysin O
CBT: cognitive behavioral therapy
CSF: cerebrospinal fluid
DSM‐5: Diagnostic and Statistical Manual of Mental Disorders, fifth edition
ESR: erythrocyte sedimentation rate
FLAIR: fluid‐attenuated inversion recovery
GAS: group A streptococcus ( *Streptococcus pyogenes* )
ICD‐11: International Classification of Diseases, 11th revision
IVIG: intravenous immunoglobulin
MRI: magnetic resonance imaging
OCD: obsessive‐compulsive disorder
PANDAS: pediatric autoimmune neuropsychiatric disorders associated with streptococcal infections
PANS: pediatric acute‐onset neuropsychiatric syndrome

## 1 Background

Pediatric autoimmune neuropsychiatric disorders associated with streptococcal infections (PANDAS) is a condition thought to be triggered by *Streptococcus pyogenes* (group A beta‐hemolytic streptococcus or GAS) infection typically in the form of acute pharyngitis. It is described as affecting children between 3 years of age and puberty and is characterized by a sudden onset of neuropsychiatric symptoms, particularly obsessive‐compulsive disorder (OCD) and tics [1]. It was first described in 1998 by Swedo et al., who proposed diagnostic criteria for this disorder [1]. These criteria include the presence of OCD or a tic disorder, symptom onset between the ages of 3 years and puberty, a dramatic onset or exacerbation of symptoms, a temporal relationship with *Streptococcus* infections, particularly GAS, confirmed by culture or serological evidence, and the presence of broad neurological abnormalities similar to Sydenham chorea [1, 2].

Subsequent recognition of similar acute neuropsychiatric presentations triggered by non‐streptococcal infections or noninfectious factors led to the introduction of pediatric acute‐onset neuropsychiatric syndrome (PANS), a broader diagnostic construct not restricted by age or etiology [3]. PANS represents a broader clinical entity defined by the sudden onset of OCD and/or avoidant/restrictive food intake disorder (ARFID), accompanied by additional neuropsychiatric symptoms. Unlike PANDAS, tics alone do not fulfill the primary diagnostic criteria for PANS. Furthermore, PANS does not require evidence of preceding streptococcal infection and encompasses a wider range of potential triggers, including infectious, metabolic, and autoimmune etiologies. PANDAS is therefore best conceptualized as a streptococcus‐associated subgroup within the broader PANS spectrum [4, 5].

Whether PANDAS can first present after puberty remains uncertain. Existing diagnostic criteria restrict PANDAS to prepubertal onset, although isolated case reports have described similar presentations in late adolescence and adulthood [2, 6]. Because the diagnosis of PANS is not limited by age or by streptococcal infection, post‐pubertal patients with evidence of a streptococcal‐triggered autoimmune neuropsychiatric syndrome are often classified as having PANS rather than PANDAS. Although this distinction rarely alters immediate management, it has important implications for understanding disease pathogenesis, epidemiology, biomarker development, and genetic susceptibility. This case series describes three post‐pubertal individuals, including two siblings, who developed abrupt‐onset neuropsychiatric symptoms following pharyngeal illness, thereby challenging the current age‐based diagnostic framework.

## 2 Case Presentation

### 2.1 Case 1

An 18‐year‐old female, born full‐term after an uncomplicated delivery, had no prior psychiatric or medical history. At age 16, while living abroad, she developed an upper respiratory tract infection characterized by pharyngitis and low‐grade fever that required a one‐day hospitalization. No throat culture was obtained. She completed a 10‐day course of antibiotics with full resolution of symptoms.

Approximately 1 month later, she experienced an abrupt onset of disabling motor and vocal tics, obsessive‐compulsive behaviors, and urinary incontinence. Motor tics included repetitive abdominal contractions, sniffing, and facial grimacing, which persisted during sleep. Her academic performance declined markedly, eventually preventing school attendance. She was subsequently evaluated at another institution abroad. According to the available medical reports from the outside institution, the patient underwent psychiatric evaluation and fulfilled the diagnostic and statistical manual of mental disorders‐5 (DSM‐5) criteria for OCD, with compulsive behaviors including repetitive switching of lights, repeated hand movements, and stepping rituals at doorways. Her responses during psychiatric interviews were described as largely monosyllabic, limiting adequate assessment of intrusive thoughts. Emotional lability and insomnia were also reported.

She underwent an extensive evaluation abroad for acute‐onset neuropsychiatric symptoms. Testing did not include antistreptolysin O (ASO) and anti‐deoxyribonuclease B (anti‐DNase B) titers or throat culture. Workup for infectious, metabolic, and autoimmune etiologies was unremarkable. Western blot for Borrelia was negative despite borderline screening IgM and IgG serologies, and she had no clinical features suggestive of Lyme disease. Empiric treatment with multiple antibiotic courses (azithromycin and doxycycline) yielded no benefit. Autoimmune encephalitis antibodies in both serum and cerebrospinal fluid (CSF) were negative, including anti‐N‐methyl‐D‐aspartate receptor (Anti‐NMDAR), anti‐glutamic acid decarboxylase (Anti‐GAD), anti‐α‐amino‐3‐hydroxy‐5‐methyl‐4‐isoxazolepropionic acid receptor 1/2 (anti‐AMPAR 1/2), anti‐gamma‐aminobutyric acid type B receptor (anti‐GABABR), anti‐contactin‐associated protein‐like 2 (anti‐CASPR2), anti‐leucine‐rich glioma‐inactivated 1 (anti‐LGI1), and anti‐gangliosides GM1‐4. Anti‐myelin basic protein (anti‐MBP) antibodies were detected at low titer in CSF but were considered nonspecific. Paraneoplastic panels, including anti‐Yo, anti‐Hu, anti‐melanoma‐associated antigen 1 and 2 (anti‐Ma1/Ma2), and anti‐Purkinje cell nuclear antigen 2 (anti‐PNMA2 Ma2/Ta), were negative. Computed tomography scans of the head, neck, chest, abdomen, and pelvis showed no evidence of malignancy. Multiple psychiatric medications, including clonazepam and sertraline, were ineffective. Approximately 2 years after symptom onset, because of persistent severe neuropsychiatric symptoms and progressive clinical deterioration, the patient was referred to our institution for further evaluation.

On examination at our institution, she was conscious and vitally stable. She exhibited hand‐clenching, poor eye contact, facial grimacing, motor tics, and simple vocalizations, including humming and hissing. Her Yale–Brown Obsessive Compulsive Scale (Y‐BOCS) score was consistent with severe OCD symptomatology. At follow‐up visits, she became largely mute with features suggestive of catatonia. Neurological examination revealed symmetric cogwheel rigidity, bradykinesia, and normal reflexes without sensory or cerebellar deficits.

At our institution, blood, urine, and throat cultures, including GAS, were negative. During periods of symptom exacerbation, serial ASO titers demonstrated an increase from 102 IU/mL to 443 IU/mL. The anti‐DNase B titer was also elevated at 502 IU/mL, erythrocyte sedimentation rate (ESR) was markedly elevated at 102 mm/h, and antinuclear antibody (ANA) was weakly positive at a titer of 1:80. Brain magnetic resonance imaging (MRI) demonstrated preserved basal ganglia structures with a small nonspecific right frontal white matter fluid‐attenuated inversion recovery (FLAIR) hyperintensity (Figure 1). These findings, together with the abrupt onset and fluctuating course of neuropsychiatric symptoms temporally associated with recurrent pharyngeal infections, raised concern for a post‐streptococcal autoimmune neuropsychiatric disorder consistent with a PANDAS‐like presentation.

**FIGURE 1:**
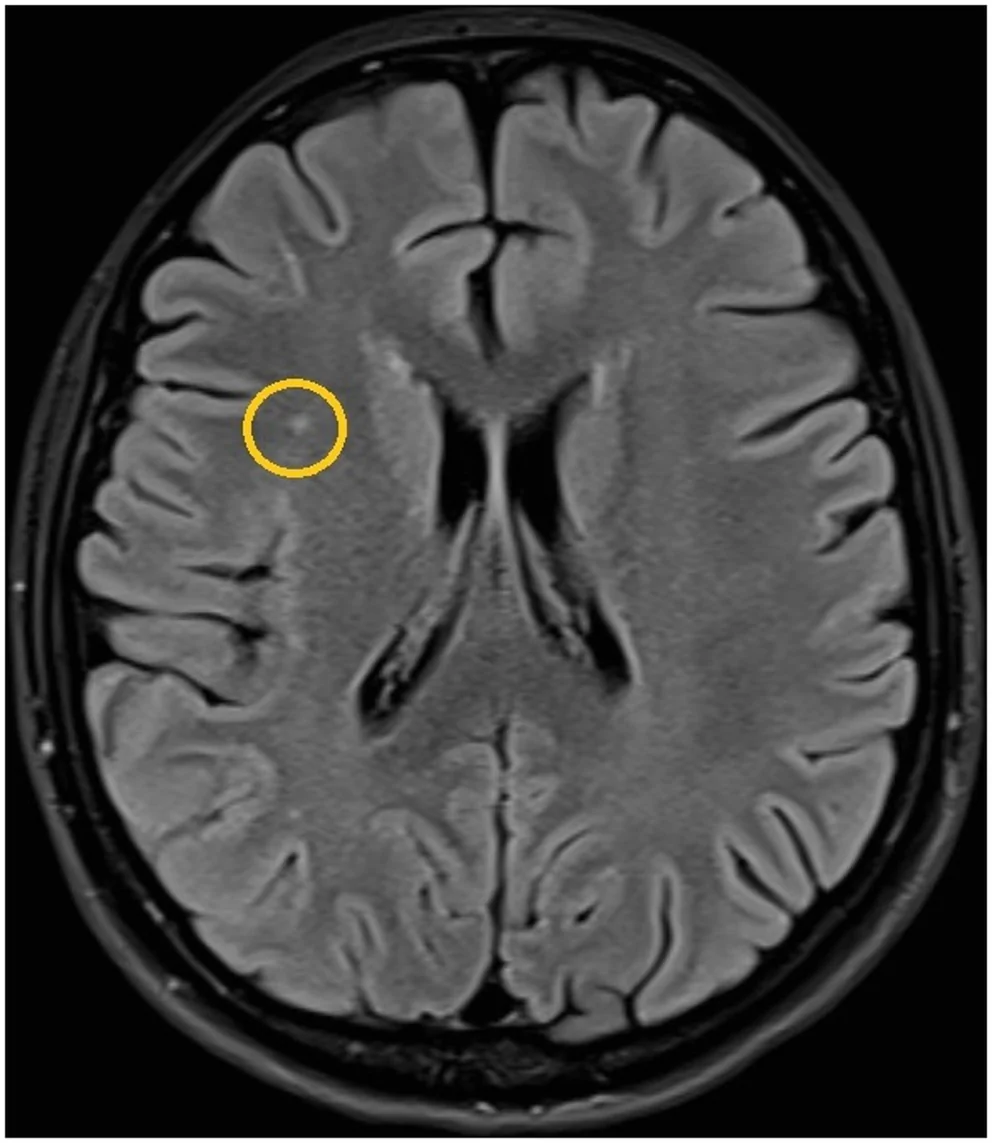
Axial brain magnetic resonance imaging (MRI), fluid‐attenuated inversion recovery (FLAIR) sequence from Case 1, demonstrating a small nonspecific hyperintense white matter lesion (yellow circle) in the right frontal periventricular/subcortical region. No associated diffusion restriction or susceptibility blooming was identified.

Initial management included sequential empiric antimicrobial therapy with amoxicillin, doxycycline, and azithromycin to provide coverage for GAS infection and other potential infectious triggers implicated in symptom exacerbation or relapse in PANDAS/PANS, including *Mycoplasma pneumoniae* , *Borrelia burgdorferi* , and *Bartonella henselae* [4, 7, 8, 9, 10, 11]. In addition, corticosteroids, as a first‐line immunomodulatory treatment, were administered to suppress possible autoimmune processes. She also received multiple psychotropic medications, including escitalopram, risperidone, and valproic acid, without meaningful clinical improvement. A mild response was observed with hydroxyzine, which resulted in some reduction in tics and improved calmness.

Because of persistent severe neuropsychiatric symptoms and limited response to antimicrobial, psychiatric, and corticosteroid therapy, escalation to additional immunomodulatory treatment was pursued. She received intravenous immunoglobulin (IVIG) initially every 4 weeks, then every 3 weeks, and ultimately weekly, completing a total of 12 sessions. Each IVIG session produced consistent but short‐lived improvement lasting approximately 5–7 days. Plasmapheresis was subsequently initiated as next‐line therapy. Six sessions resulted in marked temporary improvement, including restored mobility, social engagement, and normalized urinary continence, lasting 1 month. A second six‐session course produced only minimal and transient benefit.

### 2.2 Case 2

A 21‐year‐old male, the older brother of Case 1, was also born full‐term with an unremarkable early medical history. At age 20, 1 month after a self‐limited febrile throat infection, he abruptly developed urinary incontinence, vocal tics characterized by repetitive utterance of a single word, and severe restrictive eating behavior. He progressively refused food intake because of a fixed belief that his food was poisoned (ARFID), resulting in approximately 10 kg of weight loss. He also exhibited persecutory delusions, inappropriate affect, and generalized irrational fears.

On examination at our institution, he was conscious and oriented. He exhibited psychomotor abnormalities, including abrupt, repetitive touching of his abdomen and uncoordinated voluntary palmar movements consistent with tics. Speech was coherent but characterized by alogia. Affect was incongruent, with inappropriate smiling and fearful responses. He demonstrated generalized, persistent irrational fears and was psychotic, with frequent agitation driven by persecutory delusions. Neurological examination revealed cogwheel rigidity in both upper limbs and bradykinesia, with intact reflexes and no pathological signs, sensory deficits, or disturbances of balance or coordination. Respiratory, cardiovascular, and abdominal examinations were unremarkable.

At our institution, he underwent evaluation for autoimmune disorders, Wilson disease, and PANDAS/PANS. Blood, urine, and throat cultures, including GAS, were negative. ASO titer was 190 IU/mL, and ANA was mildly positive at a titer of 1:80. CSF analysis was unremarkable, and brain MRI revealed no abnormalities (Figure 2).

**FIGURE 2:**
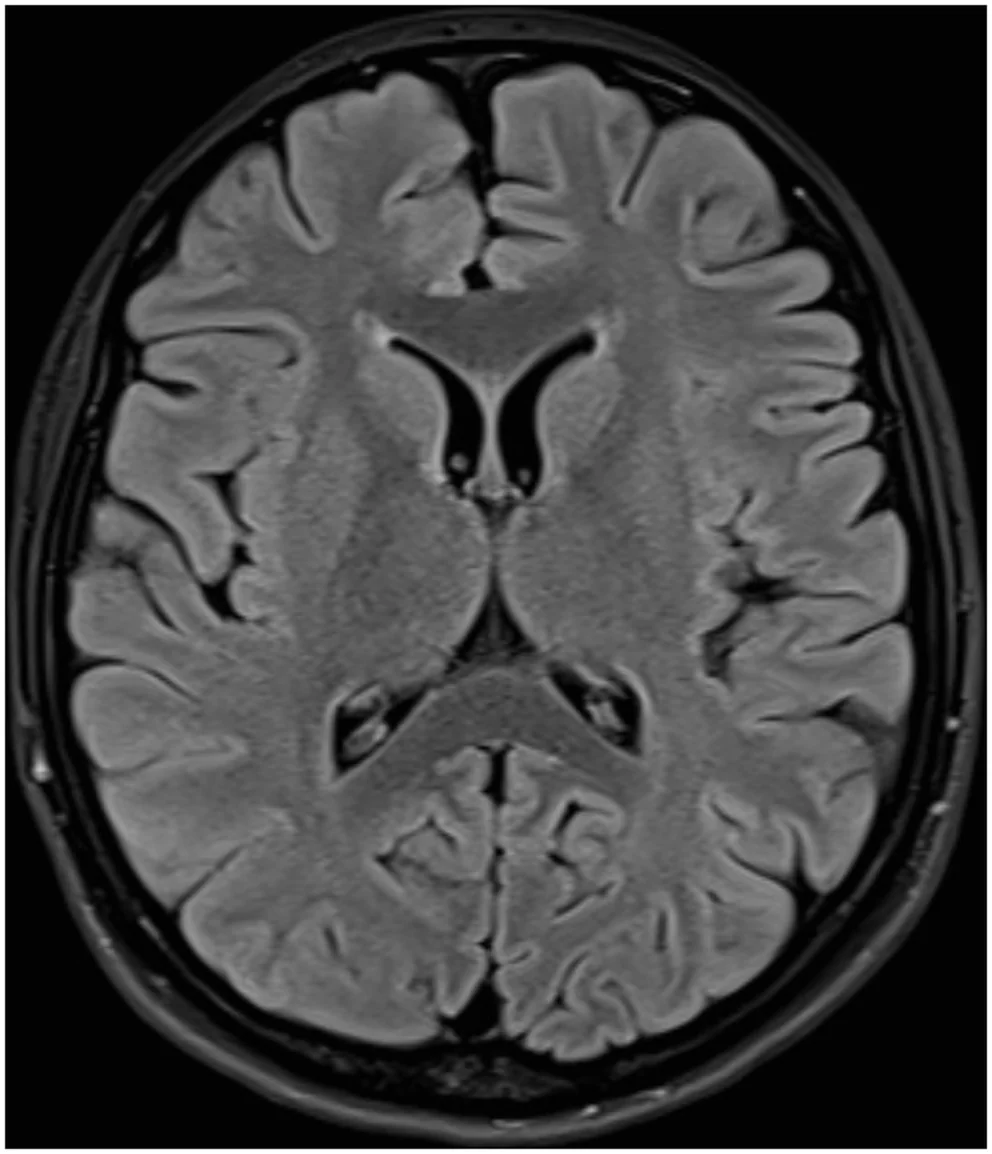
Axial brain magnetic resonance imaging (MRI), fluid‐attenuated inversion recovery (FLAIR) sequence from Case 2, demonstrating no significant intracranial abnormalities.

He was initially treated with amoxicillin, doxycycline, and azithromycin to address any potential residual infection. Because of his strong refusal to take oral medications, azithromycin was administered intravenously. He also received corticosteroids along with several psychotropic medications, including escitalopram, risperidone, and valproic acid; however, these interventions resulted in no significant clinical improvement. Consequently, his management was escalated to immunomodulatory therapy. He received four sessions of IVIG, resulting in marked improvement across all symptom domains, with clinical benefit sustained for approximately 6 months.

Before enrolling in university, he experienced an acute relapse similar to his initial presentation. Throat culture at the time of relapse was negative despite a recent febrile illness. A course of amoxicillin was administered. A second IVIG course was recommended but declined. He was also advised to pursue cognitive behavioral therapy, which he did not attend.

### 2.3 Case 3

A previously healthy 16‐year‐old male developed an acute sore throat and fever, which was treated with paracetamol. Three days later, he experienced a sudden onset of repetitive, rapid, involuntary head movements characterized by flexion–extension. Initial evaluation at another institution included a contrast‐enhanced brain CT scan, which was unremarkable. He was subsequently prescribed quetiapine 25 mg daily and escitalopram 10 mg daily.

Within 3 days of initiating these medications at the outside institution, he developed auditory hallucinations, persecutory delusions, and abnormal behavior. Over the subsequent 3 months, his condition progressively deteriorated, with the development of mutism and social withdrawal. Although he experienced severe functional regression and required assistance with most activities of daily living, he remained fully conscious and retained intermittent periods of preserved attention and motor coordination sufficient for limited engagement in simple smartphone games despite ongoing motor and vocal tics. His involuntary movements subsequently progressed to continuous multidirectional head jerks, and he developed fixed hand‐flexion posturing with near‐complete dependence for daily care.

At this point, the patient's father sought consultation at our hospital, where the patient was clinically diagnosed with PANDAS. ASO titers were not obtained because of the late presentation. Based on the documented clinical features, he did not fulfill current PANS diagnostic criteria because neither abrupt‐onset OCD nor ARFID was documented. Quetiapine and escitalopram were discontinued, and the patient was started on amoxicillin 500 mg orally every 8 h. Within 7 days, he showed a dramatic clinical improvement, becoming alert and communicative, with marked reduction of his abnormal movements.

Neurological examination at that time revealed that the patient was fully conscious and oriented. He exhibited repetitive involuntary head‐nodding movements and an abnormal finger‐to‐nose test characterized by erratic eye movements and repetitive nose tapping before locating the examiner's finger, and at times, he would continue tapping on his nose without touching the examiner's finger.

Amoxicillin was continued for 14 days, followed by prophylactic benzathine penicillin G (1.2 million units intramuscularly every 3 weeks). He continued to improve, with gradual resolution of head movements. These movements resolved completely after 2 years.

A 3‐year follow‐up assessment showed sustained remission of involuntary movements and normal ocular tracking during finger‐to‐nose testing. Mild residual impairment persisted: he occasionally tapped his nose multiple times before contacting the examiner's finger and, at times, continued tapping without reaching the target.

Table 1 summarizes the main clinical features, investigations, treatments, and outcomes of the three patients.

**TABLE 1:** Summary of clinical characteristics, investigations, treatment, and outcomes of the three post‐pubertal patients with PANDAS‐like presentations.

Feature	Case 1 (18‐year‐old female)	Case 2 (21‐year‐old male)	Case 3 (16‐year‐old male)
Triggering illness	Pharyngitis with low‐grade fever; throat culture not obtained	Febrile throat infection	Sore throat with fever
Latency to symptom onset	~1 month	~1 month	3 days
Initial neuropsychiatric symptoms	Abrupt‐onset motor and vocal tics, OCD behaviors, urinary incontinence, emotional lability	Vocal tics, urinary incontinence, food refusal, persecutory delusions	Involuntary head movements; later hallucinations, persecutory delusions, mutism
Course and severity	Progressive, severe, and disabling; periods of mutism and catatonia	Acute‐onset psychosis with agitation; persistent tics and delusions	Progressive deterioration over 3 months with severe functional impairment
Neurological findings	Cogwheel rigidity, bradykinesia; reflexes preserved	Cogwheel rigidity, bradykinesia; reflexes preserved	Continuous head‐nodding movements; abnormal finger‐to‐nose testing
Psychiatric findings	Severe OCD (Y‐BOCS score of 25), disorganized behavior, emotional lability	Psychosis with persecutory delusions, alogia, inappropriate affect	Psychosis, mutism, social withdrawal
Microbiologic tests	Throat cultures negative (late presentation); ASO increased from 102 → 443 IU/mL (normal < 200 IU/mL); anti‐DNase B 502 IU/mL (normal < 300 IU/mL)	Throat cultures negative (late presentation); ASO 190 IU/mL (normal < 200 IU/mL)	Throat culture not obtained (late presentation)
Inflammatory/autoimmune markers	ESR 102 mm/h (normal < 20 mm/h); ANA 1:80 (normal negative or < 1:40)	ANA 1:80 (normal negative or < 1:40)	Not available
CSF findings	Normal routine studies; anti‐MBP antibodies positive at low titer (nonspecific)	Completely normal	Not performed
Neuroimaging	Brain MRI: preserved basal ganglia; nonspecific right frontal FLAIR hyperintensity	Brain MRI normal	Brain CT normal
Initial treatments abroad	Multiple antibiotics and psychotropic medications; no clinical improvement	None prior to presentation; no psychiatric history	Quetiapine and escitalopram (clinical deterioration)
Antibiotic therapy at our institution	Amoxicillin, doxycycline, azithromycin	Amoxicillin, doxycycline, intravenous azithromycin	Amoxicillin (marked improvement within 7 days)
Immunomodulatory therapy	IVIG (12 courses; transient benefit); plasmapheresis (two courses of six sessions each; temporary improvement)	IVIG (four courses; marked improvement lasting approximately 6 months)	None (rapid response to antibiotics alone)
Other treatments	Corticosteroids; SSRIs; antipsychotics; valproate; limited response to hydroxyzine	Corticosteroids; SSRIs; antipsychotics; valproate	Benzathine penicillin prophylaxis every 3 weeks
Best clinical response	Partial clinical improvement after IVIG lasting 5–7 days; greatest improvement after the first plasmapheresis course, lasting approximately 1 month before relapse	Marked improvement after IVIG with sustained benefit for approximately 6 months	Marked clinical improvement within 7 days of amoxicillin therapy, with restoration of normal daily functioning within approximately 3 months
Clinical status at last follow‐up (duration of follow‐up)	Persistent relapsing course with transient responses to immunomodulatory therapy (follow‐up: 3 years)	Clinical relapse approximately 6 months after IVIG; declined further immunotherapy (follow‐up: 3 years)	Sustained clinical remission with only minimal residual motor signs after 3 years of follow‐up

Abbreviations: ANA, antinuclear antibody; ASO, antistreptolysin O; CSF, cerebrospinal fluid; CT, computed tomography; ESR, erythrocyte sedimentation rate; FLAIR, fluid‐attenuated inversion recovery; IVIG, intravenous immunoglobulin; MBP, myelin basic protein; MRI, magnetic resonance imaging; OCD, obsessive‐compulsive disorder; SSRIs, selective serotonin reuptake inhibitors; Y‐BOCS, Yale–Brown Obsessive Compulsive Scale.

## 3 Discussion

Despite increasing clinical recognition, PANDAS remains a controversial diagnosis because of heterogeneous study designs, inconsistent biomarker findings, and variable therapeutic responses. Although PANDAS is not recognized as a distinct entity in the World Health Organization's International Classification of Diseases, 11th Revision (ICD‐11), it may conceptually fall within autoimmune disorders affecting the central nervous system (8E4A.0). However, universally accepted diagnostic criteria and confirmatory laboratory tests remain unavailable; therefore, diagnosis relies primarily on clinical presentation and temporal association with preceding streptococcal infection.

The true prevalence of PANDAS remains uncertain. Early estimates suggested that up to 10% of children diagnosed with OCD or tic disorders might meet criteria for PANDAS, while more recent data from the PANS Research Consortium estimate a prevalence of approximately 1%–2% in the pediatric population [12]. Data from the Middle East are particularly sparse, with only a limited number of published case reports [13, 14], highlighting the need for regional contributions to the literature.

The underlying pathophysiology of PANDAS is hypothesized to involve a type II hypersensitivity reaction triggered by molecular mimicry between streptococcal antigens and host neuronal tissue, particularly within the basal ganglia [15]. Antineuronal antibodies elicited by GAS infection may cross‐react with host structures, resulting in neuroinflammation and neuropsychiatric manifestations, through a mechanism analogous to acute rheumatic fever [15]. However, this hypothesis remains controversial, as attempts to consistently identify specific pathogenic antibodies in patients with PANDAS have produced inconclusive and nonspecific findings [11, 16]. Accurate diagnosis requires careful exclusion of alternative overlapping neurological and psychiatric conditions, including Sydenham chorea, Tourette syndrome, primary OCD, autoimmune encephalitis, and neuropsychiatric lupus.

A particular challenge in this series is that all patients fulfilled most of Swedo's diagnostic criteria for PANDAS but did not meet the requirement for prepubertal onset, raising the question of whether PANDAS should be considered exclusively a childhood disorder. The strength of evidence for a streptococcal trigger also differed among the cases: Case 1 had rising ASO and elevated anti‐DNase B titers during symptom exacerbation, whereas Case 2 had a history of a febrile throat illness but a negative throat culture and an ASO titer within the stated reference range, possibly due to late presentation. Case 3 had a close temporal association with sore throat and fever, but no ASO testing or throat culture was obtained. Although PANDAS was originally described in children, similar post‐streptococcal neuropsychiatric syndromes have also been reported in adolescents and adults, although published cases remain limited [6, 10, 17, 18]. Several case reports and small case series have described abrupt‐onset OCD symptoms, tics, psychosis, and other neuropsychiatric manifestations temporally associated with streptococcal infections in post‐pubertal individuals, supporting the possibility that susceptibility may extend beyond childhood. Reported adult cases include a 19‐year‐old female with severe exacerbation of Tourette syndrome following streptococcal infection [19] and a 28‐year‐old male with a classical PANDAS‐like presentation [6]. However, both individuals had pre‐existing psychiatric disorders, which may complicate diagnostic interpretation. In contrast, our patients had no prior psychiatric history and presented with abrupt‐onset neuropsychiatric manifestations temporally associated with preceding pharyngeal infections, thereby strengthening the clinical suspicion for a post‐streptococcal autoimmune neuropsychiatric process.

The limited literature describing post‐pubertal PANDAS‐like presentations may partly reflect evolving diagnostic practices rather than the true rarity of the condition. Older adolescents and adults presenting with abrupt‐onset OCD symptoms, tics, restrictive eating behaviors, or other acute neuropsychiatric manifestations temporally associated with infection may preferentially be diagnosed with PANS rather than PANDAS because they no longer satisfy the strict prepubertal onset criterion required for PANDAS classification. Consequently, some post‐pubertal cases with clinical features strongly suggestive of a streptococcal‐triggered autoimmune neuropsychiatric process may be underrecognized or categorized within the broader PANS spectrum despite evidence supporting a possible post‐streptococcal etiology.

We recognize that all three patients could also be classified within the broader PANS spectrum, and that current therapeutic recommendations for PANS and PANDAS are largely similar. Nevertheless, distinguishing a probable streptococcal‐triggered subgroup remains clinically and scientifically relevant. Identifying patients with a temporal relationship to group A streptococcal infection may facilitate future studies investigating pathogen‐specific immune mechanisms, genetic susceptibility, biomarkers, prognosis, and preventive strategies, including the potential role of antimicrobial prophylaxis. Therefore, our intent is not to redefine these patients as having definitive PANDAS despite their post‐pubertal onset, but rather to highlight that streptococcal‐associated autoimmune neuropsychiatric syndromes resembling classical PANDAS may extend beyond childhood and warrant further investigation.

Familial clustering observed in some patients, including sibling involvement in our series, further raises the possibility of underlying genetic or immunologic susceptibility [20, 21]. Previous studies have demonstrated increased rates of autoimmune diseases, tic disorders, and OCD symptoms among first‐degree relatives of affected individuals, suggesting a potential hereditary contribution to disease susceptibility [1, 9]. Notably, the two siblings in our series developed the disorder at different ages, one in early adolescence and the other at 20 years, representing a familial pattern not previously reported in the literature and supporting a potential genetic predisposition. This observation parallels the well‐established genetic predisposition seen in acute rheumatic fever, in which monozygotic twins have a six‐fold increased risk when a co‐twin is affected, with heritability estimates approaching 60% [22]. Furthermore, meta‐analytic data have identified specific genetic loci associated with susceptibility to rheumatic fever [23]. Given the probable similarities in pathogenesis between PANDAS and rheumatic fever, these findings suggest that genetic factors may also contribute to the development of PANDAS.

Treatment strategies for suspected PANDAS/PANS typically involve a multidisciplinary and individualized approach that may include psychiatric and behavioral interventions, antimicrobial therapy when infection is identified or strongly suspected, anti‐inflammatory treatment, supportive care, and immunomodulatory therapies in selected moderate‐to‐severe or refractory cases [11, 17, 24]. Current consensus recommendations from the PANS Research Consortium emphasize the importance of addressing both the underlying inflammatory process and the associated neuropsychiatric manifestations through coordinated medical, psychiatric, behavioral, and psychosocial management [8, 11, 17]. Psychiatric and behavioral interventions may include cognitive behavioral therapy (CBT), psychoeducation, family support, school accommodations, and pharmacologic treatment of OCD symptoms, anxiety, mood disturbances, or psychosis when clinically indicated. Anti‐inflammatory therapies such as nonsteroidal anti‐inflammatory drugs and corticosteroids may also provide symptomatic benefit during acute exacerbations in selected patients. In our series, cognitive behavioral therapy was recommended for Case 2 as part of the comprehensive management strategy.

Immunomodulatory therapies such as corticosteroids, intravenous immunoglobulin (IVIG), and plasmapheresis are typically reserved for severe or treatment‐refractory PANDAS/PANS. Their efficacy remains controversial. Perlmutter et al. reported significant improvement with IVIG and plasmapheresis compared with placebo in children with PANDAS [25], whereas Nicolson et al. found no benefit of plasmapheresis in refractory OCD or Tourette syndrome without evidence of streptococcal infection [26]. More recently, Melamed et al. demonstrated sustained symptom improvement with repeated IVIG infusions in patients with PANS [27]. In contrast, Williams et al. found no significant benefit of IVIG over placebo in a randomized controlled trial, although participants who later received open‐label IVIG demonstrated a mean 49% reduction in OCD symptoms [28]. Responses in our patients were variable and often transient, suggesting that persistent immune activation, incomplete pathogen clearance, or host‐specific immunologic factors may influence therapeutic outcomes.

Several limitations should be acknowledged. Retrospective symptom reporting introduces the potential for recall bias, and none of the patients had microbiological confirmation of group A streptococcal infection at symptom onset. Antibody testing was incomplete in one case, and the use of multiple concurrent interventions, including antibiotics, immunomodulatory therapies, and psychotropic medications, complicates interpretation of treatment response. These limitations highlight the need for prospective studies incorporating standardized biomarkers and longitudinal assessment to better characterize post‐pubertal PANDAS‐like presentations.

In conclusion, this case series illustrates that PANDAS‐like presentations may occur in late adolescence and early adulthood and may cluster within families. While diagnostic uncertainty remains, given absent confirmatory testing and variable treatment response, the clinical pattern observed aligns with a post‐infectious neuropsychiatric syndrome temporally related to pharyngeal illness. Clinicians should consider post‐infectious autoimmune neuropsychiatric syndromes, including PANDAS‐like presentations, in older adolescents and young adults who develop abrupt‐onset OCD, tics, or acute behavioral changes following pharyngeal illness. Although many of these patients may currently be classified as having PANS, recognition of a possible streptococcal‐triggered subgroup remains important for advancing understanding of disease mechanisms, refining future diagnostic criteria, and identifying pathogen‐specific biomarkers.

## Author Contributions

**Tariq A. Madani:** conceptualization, data curation, formal analysis, investigation, methodology, project administration, resources, software, supervision, validation, visualization, writing – original draft, writing – review and editing. **Fahd S. Alsallum:** data curation, writing – original draft. **Ola H. Abudaowd:** data curation, writing – original draft. **Salman T. Madani:** data curation, writing – original draft.

## Funding

The authors have nothing to report.

## Ethics Statement

Written informed consent was obtained from all patients and their legal guardians for the publication of this case series, including clinical details. The authors confirm that patient identities have been protected, with no identifying information included in the manuscript.

## Conflicts of Interest

The authors declare no conflicts of interest.

## Data Availability

Data sharing not applicable to this article as no datasets were generated or analysed during the current study.
